# Registro SBR-AF: Preditores de Recorrência de Taquiarritmia Atrial após Primeira Ablação por Cateter na Fibrilação Atrial

**DOI:** 10.36660/abc.20240246

**Published:** 2025-01-08

**Authors:** Caique M. P. Ternes, Luis E. Rohde, Alexander Dal Forno, Andrei Lewandowski, Helcio Garcia Nascimento, Gabriel Odozynski, Claudio Ferreira, Enrico G. Ferro, Carisi A. Polanczyk, André Zimerman, Lucas S. Faganello, Eric Pasqualotto, Grazyelle Damasceno, Leandro I. Zimerman, Andre d’Avila

**Affiliations:** 1 Programa de Pós-Graduação em Cardiologia e Ciências Cardiovasculares Faculdade de Medicina Universidade Federal do Rio Grande do Sul Porto Alegre RS Brasil Programa de Pós-Graduação em Cardiologia e Ciências Cardiovasculares, Faculdade de Medicina, Universidade Federal do Rio Grande do Sul, Porto Alegre, RS – Brasil; 2 Serviço de Arritmia Cardíaca Hospital SOS Cardio Florianópolis SC Brasil Serviço de Arritmia Cardíaca, Hospital SOS Cardio, Florianópolis, SC – Brasil; 3 Divisão de Cardiologia Hospital Moinhos de Vento Porto Alegre RS Brasil Divisão de Cardiologia, Hospital Moinhos de Vento, Porto Alegre, RS – Brasil; 4 Serviço de Arritmia Cardíaca Hospital Unimed Chapecó SC Brasil Serviço de Arritmia Cardíaca, Hospital Unimed, Chapecó, SC – Brasil; 5 Harvard-Thorndike Electrophysiology Institute Beth Israel Deaconess Medical Center Harvard Medical School Boston Massachusetts EUA Harvard-Thorndike Electrophysiology Institute, Beth Israel Deaconess Medical Center, Harvard Medical School, Boston, Massachusetts – EUA; 6 TIMI Study Group Brigham and Women’s Hospital Harvard Medical School Boston Massachusetts EUA TIMI Study Group, Brigham and Women’s Hospital, Harvard Medical School, Boston, Massachusetts – EUA

**Keywords:** Fibrilação Atrial, Ablação por Cateter, Prognóstico

## Abstract

**Fundamento:**

O tratamento da fibrilação atrial (FA) com ablação por cateter evoluiu significativamente. No entanto, dados do cenário clínico real em desfechos de longo prazo são limitados, particularmente em países de baixa e média renda.

**Objetivo:**

Este estudo prospectivo, multicêntrico, do tipo coorte de pacientes consecutivos tem como objetivo avaliar a segurança da primeira ablação por cateter da FA na região sudeste do Brazil entre 2009 e 2024.

**Métodos:**

O desfecho primário foi a recorrência de qualquer taquiarritmia atrial (TAA). O modelo de riscos proporcionais de Cox avaliou preditores independentes de recorrência.

**Resultados:**

Entre 1043 pacientes (idade média 67,3 ± 11,3 anos, 27,9% mulheres), 75,5% apresentaram FA paroxística. Após um tempo mediano de acompanhamento de 1,4 (1,0 – 3,4) anos, 21,4% apresentaram recorrência de TAA. As taxas de recorrência foram de 18,6% para FA paroxística e de 29,8% para FA persistente; 67,3% dos eventos ocorreram no primeiro ano após a ablação por cateter. Preditores de recorrência foram FA persistente no basal [hazard ratio (HR) 1,57, Intervalo de Confiança (IC) 95% 1,15-2,13; p = 0,004), aumento do diâmetro atrial esquerdo (HR 1,03, IC95% 1,00-1,05; p = 0,033), e escore EHRA de sintomas de FA mais alto (HR 1,60, IC95% 1,18-2,18; p = 0,003). As taxas de recorrência diminuíram ao longo do tempo de acordo com o ano calendário do procedimento, com uma redução de 9% por ano consecutivo (HR 0,91; p < 0,001). Houve uma taxa de 2,1% de ocorrência de eventos adversos relacionados ao procedimento.

**Conclusão:**

No maior estudo coorte de ablações consecutivas da FA da América Latina, preditores de recorrência de TAA se associaram com estágios mais avançados da FA. As taxas de complicação e de recorrência foram comparáveis às de países de alta renda, destacando a aplicabilidade global da ablação por cateter para o manejo da FA.

## Graphical abstract

**Figura Central:**
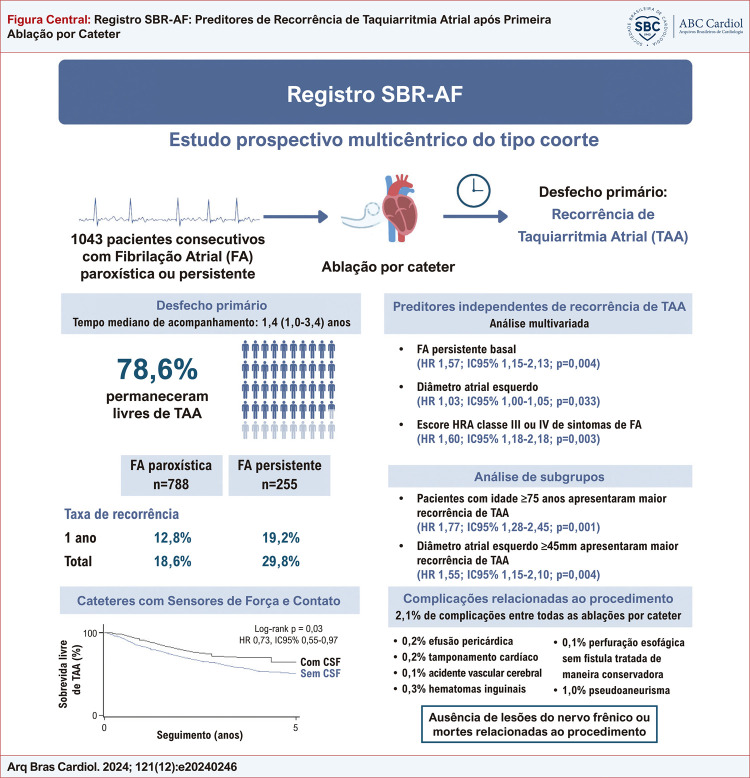
: Registro SBR-AF: Preditores de Recorrência de Taquiarritmia Atrial após Primeira Ablação por Cateter

Registro SBR-AF (Southern Brazilian Registry of Atrial Fibrillation), complicações relacionadas ao procedimento e recorrência de taquiarritmia atrial no seguimento em longo prazo.

## Introdução

A prevalência global estimada de Fibrilação Atrial (FA) foi de 44 milhões de pessoas em 2016.^1-4^ Pacientes com FA têm um risco maior de desenvolverem eventos tromboembólicos, disfunção ventricular esquerda progressiva, e pior qualidade de vida.^5,6^ Ensaios clínicos contemporâneos apoiam o controle precoce do ritmo cardíaco para melhorar os desfechos clínicos e a qualidade de vida em comparação a um controle tardio.^7,8^ A Ablação por Cateter é superior a Drogas Antiarrítmicas (DAA) em manter o ritmo sinusal e retardar a progressão da FA paroxística para FA persistente.^9,10^ Assim, a ablação por cateter tem sido cada vez mais realizada como terapia de primeira linha no controle do ritmo cardíaco em paciente com diagnóstico recente de FA.^11-13^

Apesar de evidência crescente à favor do controle do ritmo em Ensaios Clínicos Randomizados (ECRs), cenários no mundo real podem não replicar os mesmos níveis de eficácia de estudos em que centros de alto volume com operadores experientes são muito representados.^14^ Além disso, a eficácia promissora da ablação da FA precisa alinhar-se com o perfil igualmente atraente de segurança, principalmente à medida que esse procedimento é adotado por operadores e hospitais com diferentes experiência e expertise ao redor do mundo. O risco de complicações relacionadas ao procedimento pode diminuir o benefício das técnicas de ablação para controle do ritmo em pacientes com FA.^15^ Nesse sentido, muitas evidências tem se originado de ECRs e registros desenvolvidos ou em países de alta renda ou em centros de referência/acadêmicos de Países de Renda Baixa e Média (PRBM).^16-19^ No Brasil, o registro oficial mais recente sobre desfechos de ablação por cateter, financiado pela Sociedade Brasileira de Arritmias Cardíacas, é do ano de 2007.^20^ Mais recentemente, os primeiros resultados do estudo RECALL (Registro Brasileiro Cardiovascular de Fibrilação Atrial) mostraram que, no basal, somente 4,4% da população foram submetidos à ablação por cateter. Durante o seguimento, ocorreu 1,8 ablação por 100 pacientes-ano. Contudo, a eficácia da ablação por cateter não foi avaliada no estudo.^21^

Assim, a fim de esclarecer o distanciamento que existe entre as recomendações de diretrizes e dados da via real sobre o manejo da FA em PRBM, há uma necessidade urgente de se desenvolver registros estruturados para rastrear, sistematicamente, pacientes com FA, e coletar desfechos perioperatórios e de longo prazo da ablação por cateter. O estudo *Southern Brazilian Registry of Atrial Fibrillation* (SBR-AF) é atualmente o maior estudo prospectivo multicêntrico do tipo coorte da América Latina cujo objetivo é avaliar a segurança, a eficácia, e os desfechos clínicos de longo prazo de ablações consecutivas.

## Métodos

### Delineamento do estudo e critérios de eligibilidade

Conduzimos um estudo multicêntrico do tipo coorte com 1043 pacientes consecutivos com idade ≥ 18 anos, com FA paroxística, persistente, ou de longa duração, que se submeteram pela primeira vez à ablação por cateter por radiofrequência (RF) entre janeiro de 2009 e janeiro de 2024. O estudo incluiu pacientes com FA sintomática e documentada em três centros no Brasil (SOS Cardio, Florianopolis, SC; Hospital Unimed, Chapecó, SC; e Hospital Moinhos de Vento, Porto Alegre, RS).

Características basais sociodemográficas e clínicas dos participantes foram coletadas antes de cada procedimento, juntamente com o escore canadense de gravidade da FA (CCS-SAF, *Canadian Cardiovascular Society Severity of AF*) e o escore de sintomas relacionados à FA da *European Heart Rhythm Association* (EHRA).^22,23^ Todos os dados foram armazenados no programa Syscardio^®^, preservando a identidade do paciente. O Comitê de Ética local aprovou o estudo, e se obteve consentimento de todos os pacientes, seguindo-se a declaração de Helsinki.

### Protocolo de procedimento e seguimento

**Figura 1:**
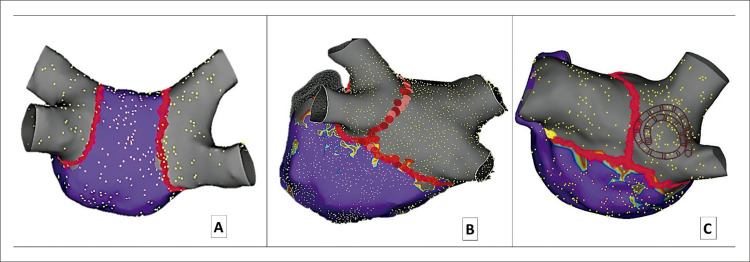
– Exemplos ilustrativos da abordagem da ablação utilizada durante o período do estudo. Somente isolamento da veia pulmonar foi realizada nos pacientes com fibrilação atrial paroxística (A). Nos demais pacientes com fibrilação atrial não paroxística, a parede posterior foi também isolada quando áreas de baixa voltagem estavam presentes em ritmo sinusal ou nos casos em que a cardioversão não pôde ser realizada com sucesso (B). Nos pacientes com um tronco comum esquerdo (C), realizou-se ablação da parede posterior do átrio esquerdo de acordo com o tipo de fibrilação atrial. Os pontos vermelhos representam a lesão da ablação. Áreas em cinza representam a ausência de atividade elétrica após a ablação, ao passo que áreas em rosa representam voltagem atrial normal em ritmo sinusal.

Todos os pacientes foram submetidos à ablação por cateter de RF sob anestesia geral. Todos os procedimentos foram realizados com diferentes versões de um *EnSite Navx - Abbott*^*®*^*.* A Figura 1 ilustra a abordagem de ablação para FA paroxística e não paroxística. Em resumo, foi realizado somente o isolamento das veias pulmonares (IVP) em pacientes com FA paroxística, enquanto a parede posterior foi incluída na maioria dos pacientes com FA não paroxística. Nos pacientes com FA paroxística, a parede posterior também foi isolada traçando-se uma linha oposta ao esôfago, quando as temperaturas desse órgão eram consideradas arriscadas para se realizar IVP. A parede posterior foi incluída em todos os pacientes com áreas de baixa voltagem em ritmo sinusal ou quando o ritmo sinusal não foi restaurado após três tentativas de cardioversão. Nos pacientes em que as áreas de baixa voltagem não estavam presentes, a ablação da parede posterior do átrio esquerdo foi realizada a critério do operador. A temperatura esofágica foi monitorada continuamente em todos os casos usando sensores Circa®, e ablação foi imediatamente interrompida se a temperatura esofágica excedesse 38^o^C. Após junho de 2016, os procedimentos de ablação foram realizados utilizando-se um cateter com sensor de força e contato. RF foi aplicada por 8-12 segundos ao longo da parede posterior e por 15-30 segundos nos demais locais, com uma corrente variando entre 650 e 700 *m*Amperes. Quando disponível, essa abordagem tipicamente resultaria em um índice de ablação de 3,5-4 para a parede posterior e de 4,5 a 5,5 ao longo da parede anterior e topo do átrio esquerdo. Infusão de isoproterenol (até 20 mcg/minuto) ou de adenosina foi usada a critério do operador até 2018. A detecção de bloqueio bidirecional da veia pulmonar (VP) e da parede posterior (quando realizada) foi o marco final do procedimento. Após a ablação por cateter, os pacientes foram mantidos em drogas antiarrítmicas por 30 dias. A amiodarona foi prescrita ou mantida para pacientes com fração de ejeção ventricular esquerda (FEVE) ≤40% e/ou doença arterial coronariana. Para pacientes com uma FEVE normal, foi prescrito 25 mg de metroprolol uma vez ao dia e 150 mg de profanenona duas vezes ao dia. Medicamentos anticoagulantes foram recomendadas por no mínimo três meses. Após os três meses iniciais, a anticoagulação oral foi usada como função do escore CHA_2_DS_2_-VASc, mas realizada a critério do médico. O acompanhamento foi conduzido por visitas pessoais 30, 160 e 360 dias após a ablação. Subsequentemente, os pacientes foram indicados a visitas anuais. Quando o paciente não pôde estar presente nas visitas anuais, outros contatos por telefone foram feitos durante o período do estudo, usando um questionário pré-definido para avaliar sintomas de arritmia. Quando casos de arritmia sintomática eram identificados em contatos telefônicos, os pacientes foram solicitados a apresentarem um eletrocardiograma e se submeterem a um exame Holter 24 horas.

### Desfecho primário

O desfecho primário foi definido como a recorrência de qualquer Taquiarritmia Atrial (TAA) avaliada por um ECG, exame de Holter ou teste de estresse cardíaco mostrando FA ou flutter atrial. Eventos que ocorreram em menos de 60 dias após o procedimento índice não foram incluídos na análise.^13^ Os pacientes foram contabilizados no último contato disponível, seja por telefone ou por escore, e considerados livres de TAA se nenhum registro de arritmia tiver sido feito após a ablação por cateter.

### Análise estatística

Os dados foram expressos em média ± desvio padrão, mediana e Intervalo Interquartil (IIQ), ou números absolutos e porcentagens. A normalidade dos dados foi avaliada usando o teste de Shapiro-Wilk, e as variáveis foram consideradas com distribuição normal quando apresentaram um valor de p > 0,05. Comparações entre os grupos (com e sem recorrência de TAA) foram realizadas pelo teste t de Student para variáveis com distribuição normal ou os testes de Wilcoxon-Mann-Whitney e de Kruskal-Wallis para variáveis sem distribuição normal. O teste do qui-quadrado foi usado para avaliar a significância da associação entre frequências das variáveis. Preditores univariados de eventos arrítmicos recorrentes (valor de p <0,10) e características basais foram avaliados com o modelo de riscos proporcionais de Cox. Valores médios foram inseridos em caso de valores faltantes de Índice de massa corporal (IMC), taxa de filtração glomerular (TFG), e diâmetro do átrio esquerdo (AE) para permitir um modelamento estatístico adequado para análise multivariada. Pacientes com FA persistente de longa duração foram incluídos no grupo de pacientes com FA persistente. Análises de sobrevida foram realizadas usando curvas de Kaplan-Meier e o teste de log-rank. Todas as análises estatísticas foram realizadas usando o Stata (versão 18). Um p-valor bicaudal de 0,05 foi considerado estatisticamente significativo.

## Resultados

### Características dos pacientes

De janeiro de 2009 a janeiro de 2024, 1043 pacientes foram submetidos pela primeira vez à ablação por cateter por FA paroxística (n=788), FA persistente (n=230), e FA persistente de longa duração (n=25). A idade média foi de 67,3 ± 11,3 anos, e 27,9% eram mulheres. A maioria dos pacientes (79,0%) relataram sintomas relacionados à FA, com 23,8% classificados com escore EHRA classe III ou IV. A maioria dos pacientes apresentou escore CHA_2_DS_2_-VASc ≥2, e 79,1% usavam anticoagulantes. A Tabela 1 descreve características clínica basais dos pacientes, estratificados por recorrência de TAA durante o acompanhamento.

**Tabela 1:** – Características basais dos pacientes com fibrilação atrial submetidos à ablação por cateter pela primeira vez

Características clínicas	Todos (n=1043)	Sem TAA (n = 820)	Recorrência de TAA (n = 223)	Valor p
Idade (anos), média ± DP	67,3 ± 11,3	66,8 ± 11,3	69,0 ± 11,2	0,01
Masculino, n (%)	752 (72,1)	598 (72,9)	154 (69,1)	0,25
Caucasiano, n (%)	1,027 (98,5)	806 (98,3)	221 (99,1)	0,10
IMC (Kg/m^2^), média ± DP	27,8 ± 4,1	27,8 ± 4,0	27,7 ± 4,3	0,91
**História e comorbidades, n (%)**				
Hipertensão	578 (55,4)	452 (55,3)	126 (55,8)	0,80
Diabetes mellitus	162 (15,5)	124 (14,9)	38 (17,4)	0,42
Doença arterial coronariana	126 (12,1)	99 (12,2)	27 (11,6)	0,96
AVC ou AIT prévio	50 (4,8)	34 (4,1)	16 (7,0)	0,07
História familiar de FA	137 (13,1)	110 (13,4)	27 (12,4)	0,59
Cardioversão elétrica prévia	529 (50,7)	404 (48,5)	125 (57,4)	0,08
Sangramento prévio	24 (2,3)	18 (2,0)	6 (3,1)	0,67
**Tipo de FA, n (%)**				<0,001
Paroxística	788 (75,5)	641 (79,0)	147 (65,1)	
Persistente	255 (24,5)	179 (21,0)	76 (34,9)	
**Escore EHRA de sintomas de FA, n (%)**				0,003
Classe I	219 (21,0)	188 (22,9)	31 (15,1)	
Classe II	576 (55,2)	451 (55,4)	125 (54,7)	
Classe III-IV	248 (23,8)	181 (21,7)	67 (30,2)	
**Escore CCS-SAF de sintomas, n (%)**				0,002
Classe 0	129 (12,4)	110 (13,2)	19 (9,7)	
Classe 1-2	578 (55,4)	469 (57,7)	109 (48,4)	
Classe 3-4	336 (32,2)	241 (29,1)	95 (41,9)	
**CHA_2_DS_2_-VASc, n (%)**				0,07
Mediana (Q1, Q3)	2 (1, 3)	2 (1, 3)	2 (1, 3)	
0-1	460 (44,1)	376 (45,5)	84 (40,0)	
2	222 (21,3)	168 (20,5)	54 (23,6)	
3	145 (13,9)	108 (13,0)	37 (16,7)	
4	83 (8,0)	65 (7,9)	20 (8,1)	
≥ 5	48 (4,6)	37 (4,6)	11 (4,6)	
**Medicamentos, n (%)**				
Amiodarona	666 (63,8)	511 (62,4)	155 (68,2)	0,07
Betabloqueadores	537 (51,5)	413 (50,3)	124 (55,0)	0,17
Aspirina	108 (10,3)	76 (8,5)	32 (15,9)	0,03
Diuréticos	152 (14,6)	106 (12,5)	46 (20,9)	0,004
**Anticoagulação, n (%)**	825 (79,1)	636 (76,9)	189 (85,6)	<0,001
Varfarina	157 (15,0)	102 (11,8)	55 (24,8)	<0,001
DOACs	668 (64,0)	534 (65,1)	134 (60,9)	<0,001
**Exames, mediana (Q1, Q3)**				
FEVE, %	64 (57-69)	65 (57-69)	63 (56-70)	0,97
Diâmetro do AE, mm	40 (36-43)	40 (36-43)	40 (37-45)	0,02
Creatinina, mg/dL	1,0 (0,9-1,2)	1,0 (0,9-1,2)	1,0 (0,9-1,1)	0,93
TFG (mL/min/1,73 m^2^)	78 (66-88)	78 (65-88)	78 (66-89)	0,80

AVC: acidente vascular cerebral; AIT: ataque isquêmico transitório; FA: fibrilação atrial; TAA: taquiarritmia atrial; IMC: índice de massa corporal; creatinina (7,6% N/A); escore CHA_2_DS_2_-VASc (8,1% N/A); CCS-SAF – Canadian Cardiovascular Society Severity of Atrial Fibrillation; DOACs: anticoagulantes orais diretos; EHRA – European Heart Rhythm Association; FEVE: fração de ejeção ventricular esquerda; TFG: taxa de filtração glomerular, (7,6% N/A); Q1 e Q3, quartis (percentis 25 e 75); DP: desvio padrão.

### Características do procedimento

IVP foi realizada em todos os pacientes usando ablação por cateter de RF, com um tempo médio de fluoroscopia de 10,6 ± 7,3 minutos e dose de radiação de 93 ± 121 mSv (dados disponíveis para 639 e 622 pacientes, respectivamente). A variação anatômica da veia pulmonar esquerda (VPE) foi determinada em casos em que duas VPEs se fundiam pelo menos 10 mm antes de sua inserção no óstio comum para desembocar no AE (Figura 1), com 26,6% (n=277) dos pacientes exibindo essa característica. O isolamento da parede posterior (IPP) adjunto (199 pacientes, 19,1%) foi realizado em pacientes com FA não paroxística e naqueles pacientes com FA paroxística que apresentavam temperatura esofágica que impossibilitava o IVP.

### Acompanhamento e recorrência de TAA

**Figura 2:**
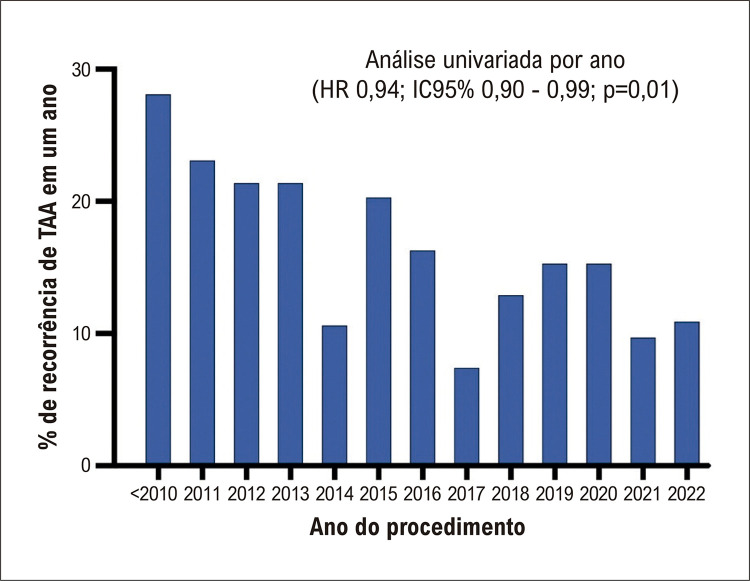
– Taxa de recorrência de Taquiarritmia Atrial (TAA) após primeira ablação por cateter segundo o ano do procedimento; IC: Intervalo de Confiança; HR: Hazard Ratio.

**Figura 3:**
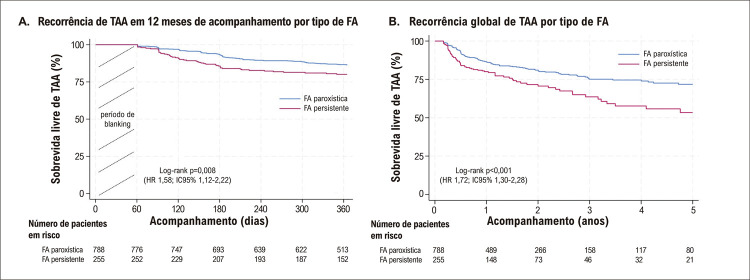
– Curva de Kaplan-Meier ilustrando a recorrência de Taquiarritmia Atrial (TAA) em pacientes com Fibrilação Atrial (FA) paroxística e não paroxística em (A) 12 meses e (B) ao final do seguimento em longo prazo; *eventos que ocorreram em menos de 60 dias após o procedimento índice não foram incluídos na análise.

**Figura 4:**
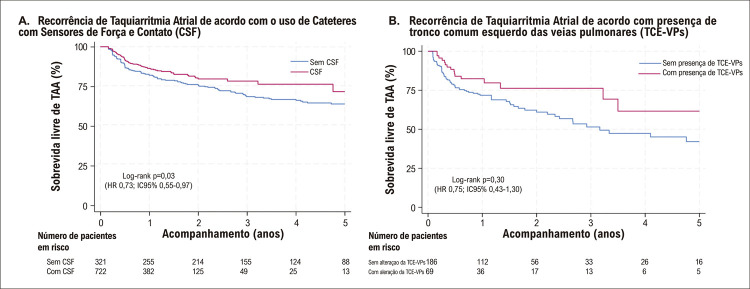
– Curvas de Kaplan-Meier para recorrência de Taquiarritmia Atrial (TAA) de acordo com (A) uso de cateteres com sensores de força e contato da primeira ablação por cateter e (B) presença de alteração anatômica de tronco comum esquerdo das veias pulmonares (TCE-VPs).

O tempo médio de acompanhamento foi de 2,5 ± 2,3 anos (mediana 1,4 [IIQ 1,0 – 3,4]. No geral, 223 (21,4%) pacientes apresentaram recorrência de TAA, 67,3% dos quais (n=150) ocorreram no primeiro ano após o procedimento. A Figura 2 ilustra a taxa de recorrência de TAA 12 meses após uma primeira ablação por cateter para FA de acordo com o ano do procedimento. Observamos uma redução gradual na recorrência de TAA na análise temporal [Hazard Ratio [HR] 0,94, intervalo de confiança (IC) de 95% de 0,90 a 0,99; p = 0,01), alcançando uma taxa reduzida após um ano de 7,5% em 2017. A Figura Suplementar 1 apresenta os números absolutos de ablações da FA e as taxas de recorrência de TAA de acordo com o ano em que o procedimento foi realizado. Pacientes com FA paroxística apresentaram uma taxa de recorrência de 12,8% em um ano e uma taxa global de 18,6%. A taxa de recorrência para paciente com FA persistente foi de 19,2% em um ano e de 29,8% durante o acompanhamento em longo prazo. Uma análise de sobrevida comparando FA paroxística *versus* FA não paroxística revelou uma maior ausência de TAA na FA paroxística como mostrado na Figura 3 tanto aos 12 meses (A) como no seguimento global. Pacientes com variação anatômica da VPE apresentaram uma liberdade de 81,6% de TAA (226/277, p=0,08). Liberdade de TAA nos pacientes com FA paroxística e não paroxística, que receberam IPP adjunto, foi de 87,2% (82/94, p = 0,11) e 77,1% (81/105, p = 0,04), respectivamente. Análise de sobrevida comparando técnicas de ablação com e sem cateteres com sensor de força e contato, ilustrada na Figura 4A, mostrou uma taxa de liberdade mais alta de TAA durante o seguimento em pacientes que se submeteram à ablação usando esses cateteres (log-rank p=0,03).

### Análise univariada e multivariada

**Figura 5:**
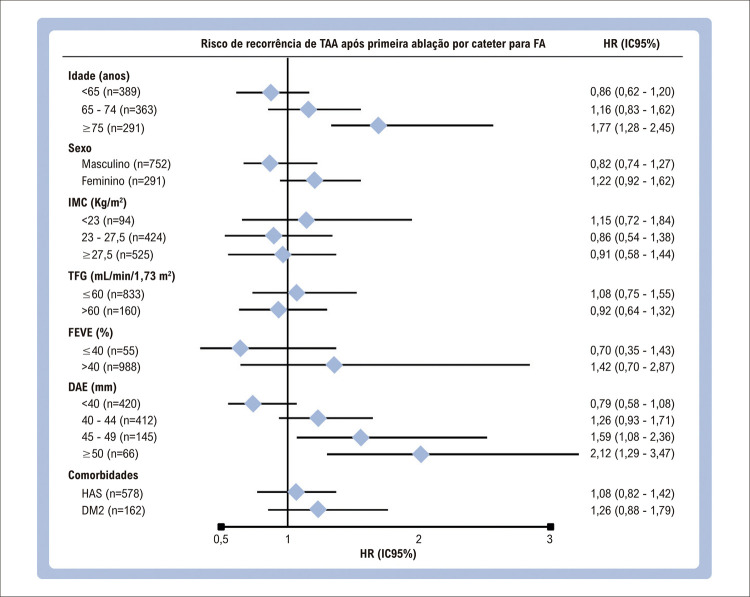
– Modelo de riscos proporcionais de Cox para o risco de recorrência de Taquiarritmia Atrial (TAA) após primeira ablação por cateter, estratificado por subgrupos; FA: fibrilação atrial; IMC: índice de massa corporal; FEVE: fração de ejeção ventricular esquerda; DAE: diâmetro átrio esquerdo; IC: intervalo de confiança; HR: hazard ratio; HAS: hipertensão; DM2: Diabetes mellitus tipo 2; TFG: taxa de filtração glomerular.

Análise univariada e modelo de riscos proporcionais de Cox para avaliar preditores de recorrência de TAA após ablação da FA estão apresentados na Tabela 2. Preditores independentes de recorrência de TAA após primeira ablação incluíram FA persistente no basal (HR 1,57; IC95% 1,15 – 2,13; p = 0,004), maior diâmetro do AE (em milímetros) (HR 1,03; IC95% CI 1,00 – 1,05; p = 0,033), e paciente com um escore EHRA classe III ou IV (HR 1,60, IC95% 1,18 – 2,18; p = 0,003) (Figura Central). O ano do procedimento foi um fator protetor independente, com uma redução relativa de 9% na recorrência para cada novo ano do calendário do programa de ablação (Figura 2 e Tabela 2). Esses achados foram consistentes em uma análise restrita a pacientes com FA paroxística, como apresentado na Tabela Suplementar 1. Uma análise de subgrupos de recorrência de TAA entre diferentes faixas etárias, sexo, categorias de IMC, hipertensão, diabetes tipo 2, TFG, fração de ejeção ventricular esquerda, e diâmetro do AE está apresentada na Figura 5. Ente os subgrupos, os pacientes com idade superior a 75 anos (HR 1,77, IC95% 1,28 – 2,45; p = 0,001) e um diâmetro atrial esquerdo mais alto (45-49mm, p = 0,020; >50mm, p = 0,003) apresentaram desfechos estatisticamente significativos em relação à TAA (HR 1,55, IC95% 1,15-2,10, p = 0,004).

**Tabela 2:** – Análise univariada e modelo de riscos proporcionais de Cox para recorrência de taquiarritmia atrial após ablação com cateter por radiofrequência

	Análise univariada			Análise multivariada		
	HR	IC95%	p	HR	IC95%	p
Ano consecutivo do procedimento	0,94	0,90 – 0,99	0,010	0,91	0,87 – 0,96	<0,001
Fibrilação atrial persistente	1,72	1,30 – 2,28	<0,001	1,57	1,15 – 2,13	0,004
Diâmetro atrial esquerdo alargado (mm)	1,03	1,01 – 1,05	0,002	1,03	1,00 – 1,05	0,033
Escore EHRA de sintomas de FA Classe III-IV	1,94	1,26 – 2,97	0,002	1,60	1,18 – 2,18	0,003
Sexo	0,82	0,62 – 1,09	0,172			
Idade	1,01	1,00 – 1,03	0,027			
Hipertensão	1,08	0,82 – 1,42	0,590			
Diabetes tipo 2	1,26	0,88 – 1,79	0,205			
AVC prévio	1,74	1,04 – 2,90	0,033			
Uso de betabloqueadores	1,20	0,92 – 1,57	0,172			
Uso de diuréticos	1,38	1,00 – 1,91	0,052			
Presença de TCE-VPs	0,91	0,66 – 1,24	0,548			
Uso de catetes com SFC	0,73	0,55 – 0,97	0,030			
Cardioversão elétrica prévia	1,28	0,96 – 1,70	0,088			

SFC: sensor de força de contato; FA: fibrilação atrial; EHRA: European Heart Rhythm Association; AVC: acidente vascular cerebral; TCE-VPs.

A análise multivariada identificou apenas a idade (HR 1,03; IC95% 1,00 – 1,05, p = 0,01) como um preditor independente de recorrência de TAA quando restrita a pacientes com FA persistente (Tabela Suplementar 2). A Figura 4B ilustra a análise de sobrevida em pacientes com FA persistente com e sem alteração da TCE-VPs (log-rank p=0,30).

### Segurança e eventos adversos

Ao longo de 15 anos, entre as 1043 ablações consecutivas realizadas, a taxa de complicação durante a admissão índice foi de 2,1% (Tabela Suplementar 3). Vale notar que os pacientes com idade superior a 75 anos representaram apenas 0,8% das complicações totais. Eventos adversos graves incluíram dois casos de tamponamento cardíaco (um caso tratado com sucesso durante o procedimento e um necessitou de cirurgia cardíaca), um acidente vascular cerebral não fatal durante a admissão para o procedimento índice, e uma perfuração esofágica sem fístula tratada com sucesso de maneira conservadora. Não foi observada lesão de nervo frênico, estenose da veia pulmonar clinicamente relevante, ou mortes relacionadas ao procedimento (Figura Central).

## Discussão

Este estudo prospectivo multicêntrico do tipo coorte avaliou mais de mil pacientes consecutivos com FA submetidos pela primeira vez à ablação no Brasil, e apresenta dados de longo prazo sobre eficiência, segurança, e preditores de recorrência de arritmia. Os principais achados da análise presente incluem: (i) eficácia e segurança global foram comparáveis às descritas em ensaios clínicos e registros de países de alta renda na Europa e na América do Norte;^14,17^ (ii) a eficácia em longo prazo aumentou ao decorrer do tempo, com uma redução de 9% no risco relativo de recorrência de TAA para cada ano consecutivo em que a ablação foi realizada; (iii) a adoção de catéteres com sensores de força e contato melhorou os desfechos após a primeira ablação; (iv) a maioria das recorrências de TAA ocorreu em um ano após a ablação; e (v) a recorrência de TAA foi mais frequentemente observada em procedimentos realizados em pacientes com doença avançada (escore de EHRA grave de sintomas de FA, AE maior e FA persistente). Além disso, nossos achados destacam a baixa taxa de complicações de ablações consecutivas em um estudo prospectivo do tipo coorte conduzido na América Latina.

Registros internacionais exercem um papel crucial em compreender disparidades entre diretrizes e o manejo da FA na prática clínica diária. Um relatório inicial da *Atrial Fibrillation Ablation Pilot Registry of the European Society* (EORP-AF) demonstrou taxas de sucesso de um ano após a ablação variando entre 69% e 74,7% em diferentes países.^24^ O EORP-AF também contribuiu significativamente para o entendimento de dados da vida real relacionados à FA.^25^ No EORP-AF Long-Term Registry, os desfechos foram documentados de 9663 pacientes com FA com base no seu tratamento antitrombótico.^26^Enquanto 42% e 33% dos pacientes do EORP-AF usaram Antagonistas de Vitamina-K (AVK) e Anticoagulantes Orais Diretos (DOAC), respectivamente, nossa coorte exibiu um padrão diferente, com 15% usando AVK e 64% DOACs. Desfechos da ablação baseados no gênero também foram relatados nessa colaboração europeia, com uma representação similar de gênero à observada em nosso estudo, onde cerca de somente 30% dos pacientes eram do gênero feminino. No basal, pacientes do sexo feminino eram mais sintomáticas em comparação ao gênero masculino, com escores EHRA médios de 2,6 vs. 2,4 na Europa (p<0,001)^27^ e 2,2 vs. 2,0 no Brasil (p<0,001). Um dado importante é que nenhum dos estudos relatou diferenças baseadas no gênero estatisticamente significativas nas taxas de recorrência em 12 meses (34,4% vs. 34,2% na Europa; 16,1% vs. 13,7% no Brasil, p=0,3), destacando a necessidade de acesso igualitário como uma opção terapêutica para as mulheres.^27^

Este estudo representa o maior estudo do tipo coorte até o momento delineado para avaliar desfechos da ablação em pacientes com FA na América Latina. Dados de pacientes brasileiros com FA foram recentemente descritos no estudo RECALL, embora desfechos de ablação não tenham sido avaliados.^21^ O último registro multicêntrico dedicado a desfechos de ablação publicado no Brasil foi conduzido pela Sociedade Brasileira de Arritmias Cardíacas entre 2005 e 2006.^20^ Nesse registro, 755 pacientes com FA foram incluídos, e uma taxa de complicação de 14,3% foi relatada, que incluiu 1,4% de eventos isquêmicos neurológicos transientes, 0,4% de estenose na veia pulmonar, 3,8% de hematomas na região inguinal, e 2,3% de outras complicações. A taxa de 2,1% de complicações observadas na coorte atual destaca a curva de aprendizagem associada com procedimentos de ablação e demonstra como avanços tecnológicos, principalmente cateteres com sensores de força e contato, tornaram esses procedimentos mais seguros e mais confiáveis na prática clínica. Resultados similares foram observados no maior estudo do tipo coorte, o *NCDR AFib Ablation Registry*, com uma taxa de complicação de 2,5% nos 76219 pacientes com FA ao longo de cinco anos.^17^

Estudos prévios abordaram preditores de recorrência após ablação da FA. A análise presente demonstra que FA persistente e um AE maior foram consistentemente relatados como fatores de risco independentes.^28,29^ Vários escores foram desenvolvidos para predizer desfechos de ritmo após ablação da FA. O escore APPLE (um ponto para idade > 65 anos, FA persistente, TFG estimada < 60 mL/min/1,73 m^2^, diâmetro do AE ≥43mm, FEVE < 50%) mostrou um desempenho subótimo (AUC = 0,64),^30^ enquanto a calculadora da web AFA-Recur, que se baseia em um modelo *random forest* de 19 variáveis alcançou um desempenho discriminatório aceitável (AUC 0,72).^31^ Nosso modelo de Cox também incorporou o escore EHRA de sintomas de FA classes III-IV como um preditor independente de risco. O escore EHRA AF é comumente usado para avaliar a resposta clínica após a ablação,^32^ e pode também sinalizar a gravidade e a duração mais longa da doença. Em comparação a registros anteriores, nossa coorte também mostrou taxas mais altas de TAA após a ablação em pacientes mais velhos, embora não houve diferença significativa nos desfechos entre as categorias de IMC.^33,34^

É razoável considerar que pacientes com FA paroxística expostos a cargas mais longas de arritmia sofram um remodelamento progressivo do átrio. Essa possibilidade leva a uma piora da atriopatia subjacente e progressão a formas mais graves e persistentes da doença. Por fim, essa progressão esperada da FA leva a piores desfechos clínicos em procedimentos realizados em estágios mais tardios da história natural da arritmia. Essa proposta foi sustentada no ensaio EARLY-AF, em que pacientes com FA paroxística foram acompanhados por três anos.^35^ Esse estudo revelou que pacientes submetidos à ablação inicial apresentaram menor progressão à FA persistente e menos recorrências de TAA em comparação a pacientes tratados somente com drogas antiarrítmicas. Embora pareça evidente que ablações mais precoces poderiam levar a melhores resultados, atingir uma eficácia elevada em ablações de FA persistente continua um desafio. Locais adjuntivos de ablação, tais como IPP, foram recentemente sugeridos como uma estratégia potencial no manejo dessa condição desafiadora.^36,37^ Em nossa coorte, o IPP foi realizado em aproximadamente um quinto dos pacientes incluídos segundo critérios do operador, mas não foi um preditor independente de recorrência de TAA.

### Pontos fortes e limitações do estudo

Nossa coorte é constituída de pacientes consecutivos com FA submetidos à ablação pela primeira vez, o que a torna o maior conjunto de dados da América Latina destinado a avaliar a segurança e a eficácia da ablação da FA. Esses achados são particularmente relevantes no contexto das alterações do TCE-VPs, fornecendo *insights* valiosos à segurança clínica no mundo real. Fatores de confusão residuais são uma preocupação potencial, uma vez que faltaram dados para o ajuste quanto à duração da FA. Não analisamos os desfechos de ablações repetidas neste estudo. Este estudo multicêntrico do tipo coorte foi conduzido somente em centros privados e pode não refletir a realidade de centros públicos no Brasil. A maioria dos pacientes eram brancos e não representaram a população da América Latina. Ainda, os pacientes foram contabilizados no último acompanhamento, o que pode ter subestimado a taxa de recorrência de TAA.

## Conclusão

No maior estudo coorte da América Latina de pacientes consecutivos com FA submetidos à primeira ablação, a recorrência de TAA foi associada a intervenções realizadas em estágios mais avançados da doença, destacando a importância da intervenção precoce para melhores desfechos clínicos. Complicações do procedimento e taxas de recorrência da TAA foram comparáveis às de países de alta renda, destacando a aplicabilidade global da ablação por cateter para o manejo da FA. Assim, esses dados reforçam o excelente desempenho da ablação no manejo da FA em centros da América Latina, sugerindo que essa opção terapêutica deveria ser expandida ao sistema público no Brasil
