# Supplementary material for: Registro SBR-AF: Preditores de Recorrência de Taquiarritmia Atrial após Primeira Ablação por Cateter na Fibrilação Atrial
Source: Arq Bras Cardiol. 2025 Jan 8;122(1):e20240246. [Article in Portuguese] doi: 10.36660/abc.20240246 (PMC11809865; doi:10.36660/abc.20240246)
Supplement: Translation

# Translation: The Southern Brazilian Registry of Atrial Fibrillation (SBR-AF Registry): Predictors of Atrial Arrhythmia Recurrence after First-Time Catheter Ablation

**DOI:** 10.36660/abc.20240246i

**Authors:** Caique M. P. Ternes, Luis E. Rohde, Alexander Dal Forno, Andrei Lewandowski, Helcio Garcia Nascimento, Gabriel Odozynski, Claudio Ferreira, Enrico G. Ferro, Carisi A. Polanczyk, André Zimerman, Lucas S. Faganello, Eric Pasqualotto, Grazyelle Damasceno, Leandro I. Zimerman, Andre d’Avila

**Affiliations:** 1 Brazil Programa de Pós-Graduação em Cardiologia e Ciências Cardiovasculares, Faculdade de Medicina, Universidade Federal do Rio Grande do Sul,Porto Alegre, RS – Brazil; 2 Brazil Serviço de Arritmia Cardíaca, Hospital SOS Cardio, Florianópolis, SC – Brazil; 3 Brazil Divisão de Cardiologia, Hospital Moinhos de Vento, Porto Alegre, RS – Brazil; 4 Brazil Serviço de Arritmia Cardíaca, Hospital Unimed,Chapecó, SC – Brazil; 5 USA Harvard-Thorndike Electrophysiology Institute, Beth Israel Deaconess Medical Center, Harvard Medical School,Boston, Massachusetts – USA; 6 USA TIMI Study Group, Brigham and Women’s Hospital, Harvard Medical School, Boston, Massachusetts – USA

**Keywords:** Atrial Fibrillation, Catheter Ablation, Prognosis

## Abstract

**Background:**

Treatment of atrial fibrillation (AF) with catheter ablation (CA) has evolved significantly. However, real-world data on long-term outcomes are limited, particularly in low- and middle-income countries.

**Objective:**

This multicenter prospective cohort of consecutive patients aimed to evaluate the safety and efficacy of first-time CA for AF in Southern Brazil from 2009 to 2024.

**Methods:**

The primary outcome was any atrial tachyarrhythmia (ATA) recurrence. Multivariable Cox proportional hazards model assessed independent predictors of recurrence.

**Results:**

Among 1,043 patients (mean age 67.3 ± 11.3 years, 27.9% female), 75.5% had paroxysmal AF. At a median follow-up of 1.4 (1.0 - 3.4) years, 21.4% had ATA recurrence. Recurrence rates were 18.6% for paroxysmal and 29.8% for persistent AF, and 67.3% of events occurred within the first year after CA. Predictors of recurrence were persistent AF at baseline (hazard ratio [HR] 1.57, 95% confidence interval [CI] 1.15-2.13; p = 0.004), enlargement of left atrial diameter (HR 1.03, 95% CI 1.00-1.05; p = 0.033), and higher EHRA score of AF symptoms (HR 1.60, 95% CI 1.18-2.18; p = 0.003). Recurrence rates decreased over time according to the procedure’s calendar year, with a 9% relative reduction per consecutive year (HR 0.91; p < 0.001). There was a 2.1% rate of procedure-related adverse events.

**Conclusions:**

In the largest cohort study of consecutive AF ablations in Latin America, predictors of ATA recurrence were related to later stages of AF. Complication and recurrence rates were comparable to those in high-income countries, underscoring the global applicability of CA for AF management.

## Introduction

The estimated global prevalence of atrial fibrillation (AF) was of 44 million people in 2016.^1-4^ AF patients are at increased risk for thromboembolic events, progressive left ventricular dysfunction, and worsening quality of life.^5,6^ Contemporary trials support early rhythm control to improve clinical outcomes and quality of life compared with late control.^7,8^ Catheter ablation (CA) of AF is superior to anti-arrhythmic drugs (AAD) in maintaining sinus rhythm and delaying progression from paroxysmal to persistent AF.^9,10^ Therefore, CA has been increasingly performed as the first-line rhythm control therapy for patients with recently diagnosed AF.^11-13^

Despite increasing evidence favoring rhythm control in randomized clinical trials (RCTs), real-world scenarios may not replicate the same levels of efficacy from studies in which high-volume centers with experienced operators are commonly overrepresented.^14^ Furthermore, the promising efficacy of AF ablation needs to be matched by an equally appealing safety profile, especially as this procedure is adopted by operators and hospitals with heterogeneous experience and expertise around the world. The risk of peri-procedural complications might reduce the overall net clinical benefit of ablation techniques for rhythm control in AF patients.^15^ In this regard, much of the evidence has been drawn from RCTs and registries conducted either in high-income countries or in reference/academic centers in low and middle-income countries (LMICs).^16-19^ In Brazil, the most recent official registry on CA outcomes sponsored by *the Brazilian Society of Cardiac Arrhythmias* dates back to 2007.^20^ More recently, the RECALL Study (*Brazilian Cardiovascular Registry of Atrial Fibrillation*) primary results showed that at baseline, only 4.4% of the population had undergone CA. During follow-up, there were 1.8 ablations per 100 patient-years. However, the effectiveness of CA was not assessed in the study.^21^

Thus, to gain a clear understanding of gaps between guideline recommendations and real-world data on AF management in LMICs, there is an urgent need for structured registries to systematically track AF patients and collect peri-operative and long-term outcomes of CA. The current Southern Brazilian Registry of Atrial Fibrillation (SBR-AF) study is the largest multicenter prospective cohort in Latin America to date dedicated to assessing the peri-procedural safety, efficacy, and long-term clinical outcomes of consecutive ablations.

## Methods

### Study design and eligibility criteria

We conducted a prospective multicenter cohort study of 1,043 consecutive patients ≥ 18 years of age, with paroxysmal, persistent, or longstanding persistent AF who underwent a first-time radiofrequency (RF) CA from January 2009 to January 2024. The study included patients with symptomatic and documented AF in 3 centers in Brazil (SOS Cardio, Florianopolis, SC; Hospital Unimed, Chapecó, SC; and Hospital Moinhos de Vento, Porto Alegre, RS).

Baseline sociodemographic and clinical characteristics were collected before each procedure, together with the Canadian Cardiovascular Society Severity of AF (CCS-SAF) score and the European Heart Rhythm Association (EHRA) score of AF-related symptoms (EHRA score).^22,23^ All data were stored in Syscardio^®^ software, preserving the patient's identity. Local Ethics Committees approved the study, and patients provided informed consent in accordance with the Helsinki Declaration.

### Procedural protocol and follow-up

All patients underwent RFCA under general anesthesia. All procedures were performed with different versions of an *EnSite Navx - Abbott®.*Figure 1 illustrates the ablation approach for paroxysmal a non-paroxysmal AF. Briefly, only pulmonary vein isolation (PVI) was performed in patients with paroxysmal AF, whereas the posterior wall was included in most patients with non-paroxysmal AF. In patients with paroxysmal AF, the posterior wall was also isolated using a posterior wall line in opposite to the esophagus when high esophageal temperatures were deemed too risky and prevented PVI. The posterior wall was included in all patients with areas of low voltage identified in sinus rhythm or when sinus rhythm was not restored after three cardioversion attempts did not restore. In those patients in whom areas of low voltage were not present, ablation of the posterior wall of the left atrium was performed according to the operator’s discretion. Esophageal temperature was continuously monitored in all cases using Circa^®^ sensors and ablation was immediately stopped if the esophageal temperature exceeded 38°C. After June 2016, ablation procedures were performed with contact-force sensing (CFS) catheters. RF applications were delivered for 8-12 seconds along the posterior wall and 15-30 seconds everywhere else, with a current ranging between 650-700 *m*Amperes. When available, this approach would typically result in an ablation index of 3.5-4 for the posterior wall and 4.5 to 5.5 along the anterior wall and roof of the left atrium. Isoproterenol (up to 20 mcg/min) or adenosine infusion was used at the operators' discretion until 2018 but not after that. Detection of pulmonary vein (PV) (all patients) and posterior wall (when performed) bidirectional block was the endpoint of the procedure. After CA, patients were kept on antiarrhythmic drugs for 30 days. Amiodarone was prescribed or continued for patients with left ventricular ejection fraction (LVEF) ≤ 40% and/or coronary artery disease. Patients with a normal LVEF were prescribed 25 mg of metroprolol one a day and 150 mg of profanenone twice a day. Anticoagulation medications were recommended for at least three months. Beyond the initial three months, oral anticoagulation was utilized as a function of the CHA_2_DS_2_-VASc score but ultimately left at the physician’s discretion. Follow-up was conducted with in-person visits at 30, 180, and 360 days after ablation. Subsequently, patients were recommended for yearly visits. Upon failure to return for a yearly visit, additional contact was made by phone contacts throughout the study period, using a pre-specified query to assess arrhythmia symptoms. In cases of symptomatic arrhythmia identified in phone contacts, patients were asked to present an electrocardiogram (ECG) and to undergo Holter monitoring.

**Figure 1:** – Illustrative examples of the ablation approach utilized during the study period. Only pulmonary vein isolation was performed in patients with paroxysmal atrial fibrillation (A). In all other non-paroxysmal patients, the posterior wall was also isolated when areas of low voltage were present in sinus rhythm or when patients could not be successfully cardioverted (B). In patients with a left common trunk (C), the posterior wall of the left atrium was ablated according to the type of atrial fibrillation. The red dots represent the ablation lesion. Areas in gray represent the absence of electrical activity after ablation whereas areas in pink represent normal atrial voltage in sinus rhythm.

### Primary outcome

The primary outcome was defined as the recurrence of any documented atrial tachyarrhythmia (ATA) assessed by either an ECG, Holter monitor, or a cardiac stress test showing AF or atrial flutter. We allowed a blanking period of 60 days, *i.e*., events occurring less than 60 days from the index procedure were not included in the current analysis.^13^ Patients were censored at the last available contact, either by phone or in-person visit, and they were considered free of ATA if no records of arrhythmia were made after CA.

### Statistical analysis

Data were expressed as mean ± standard deviation, median and interquartile range (IQR), or absolute numbers and percentages. Data normality was assessed using the Shapiro-Wilk test, and variables were considered normally distributed when their significance p-value was > 0.05. Comparisons between groups (with and without ATA recurrence) were performed using the Student’s t-test test for normally distributed variables or the Wilcoxon-Mann-Whitney and Kruskal-Wallis test for variables with non-normal distribution. The chi-square test was used to assess the significance of the association between frequencies of variables. Univariate predictors of recurrent arrhythmic events (p-value < 0.10) and baseline characteristics were evaluated with the multivariable Cox proportional hazards model. Mean values were interpolated for missing values in body mass index (BMI), glomerular filtration rate (GFR), and left atrial (LA) diameter to allow adequate statistical modeling for multivariable analysis. Longstanding persistent AF patients were included in the persistent AF group. Survival analyses were performed using Kaplan-Meier curves and the log-rank test. All statistical analyses were made using Stata (version 18). A two-tailed p-value of 0.05 was considered statistically significant.

## Results

### Patient characteristics

From January 2009 through January 2024, a total of 1,043 patients underwent first-time CA for paroxysmal (n = 788), persistent (n = 230), and longstanding persistent AF (n=25) patients. Mean age was 67.3 ± 11.3 years, and 27.9% were female. Most patients (79.0%) reported AF-related symptoms, with 23.8% classified with EHRA Score class III or IV. Most patients had CHA_2_DS_2_-VASc scores ≥ 2, and 79.1% were on anticoagulants. Table 1 describes baseline clinical characteristics of patients, stratified by ATA recurrence during follow-up.

**Table 1:** – Baseline characteristics of patients with atrial fibrillation undergoing first-time catheter ablation

Clinical Characteristic	All (n=1,043)	ATA-Free (n = 820)	ATA recurrence (n = 223)	p value
Age (years), mean ± SD	67.3 ± 11.3	66.8 ± 11.3	69.0 ± 11.2	0.01
Male, n (%)	752 (72.1)	598 (72.9)	154 (69.1)	0.25
White, n (%)	1.027 (98.5)	806 (98.3)	221 (99.1)	0.10
BMI (kg/m^2^) mean ± SD	27.8 ± 4.1	27.8 ± 4.0	27.7 ± 4.3	0.91
**History and Comorbidities, n (%)**				
Hypertension	578 (55.4)	452(55.3)	126 (55.8)	0.80
Diabetes mellitus	162 (15.5)	124 (14.9)	38 (17.4)	0.42
Coronary artery disease	126 (12.1)	99 (12.2)	27 (11.6)	0.96
Previous stroke or TIA	50 (4.8)	34 (4.1)	16 (7.0)	0.07
Family history of AF	137 (13.1)	110 (13.4)	27 (12.4)	0.59
Prior direct cardioversion	529 (50.7)	404 (48.5)	125 (57.4)	0.08
Prior bleeding	24 (2.3)	18 (2.0)	6 (3.1)	0.67
**Type of AF, n (%)**				<0.001
Paroxysmal	788 (75.5)	641 (79.0)	147 (65.1)	
Persistent	255 (24.5)	179 (21.0)	76 (34.9)	
**EHRA Score of AF Symptoms, n (%)**				0.003
Class I	219 (21.0)	188 (22.9)	31 (15.1)	
Class II	576 (55.2)	451 (55.4)	125 (54.7)	
Class III-IV	248 (23.8)	181 (21.7)	67 (30.2)	
**CCS-SAF Symptom Score, n (%)**				0.002
Class 0	129 (12.4)	110 (13.2)	19 (9.7)	
Class 1-2	578 (55.4)	469 (57.7)	109 (48.4)	
Class 3-4	336 (32.2)	241 (29.1)	95 (41.9)	
**CHA_2_DS_2_-VASc, n (%)**				0.07
Median (Q1, Q3)	2 (1, 3)	2 (1, 3)	2 (1, 3)	
0-1	460 (44.1)	376 (45.5)	84 (40.0)	
2	222 (21.3)	168 (20.5)	54 (23.6)	
3	145 (13.9)	108 (13.0)	37 (16.7)	
4	83 (8.0)	65 (7.9)	20 (8.1)	
≥ 5	48 (4.6)	37 (4.6)	11 (4.6)	
**Drugs, n (%)**				
Amiodarone	666 (63.8)	511 (62.4)	155 (68.2)	0.07
ß blockers	537 (51.5)	413 (50.3)	124 (55.0)	0.17
Aspirin	108 (10.3)	76 (8.5)	32 (15.9)	0.03
Diuretics	152 (14.6)	106 (12.5)	46 (20.9)	0.004
**Anticoagulation, n (%)**	825 (79.1)	636 (76.9)	189 (85.6)	<0.001
Warfarin	157 (15.0)	102 (11.8)	55 (24.8)	<0.001
DOACs	668 (64.0)	534 (65.1)	134 (60.9)	<0.001
**Exams, median(Q1, Q3)**				
LVEF, %	64 (57-69)	65 (57-69)	63 (56-70)	0.97
LA diameter, mm	40 (36-43)	40 (36-43)	40 (37-45)	0.02
Creatinine, mg/dl	1.0 (0.9-1.2)	1.0 (0.9-1.2)	1.0 (0.9-1.1)	0.93
GFR, ml/min/1.73 m^2^	78 (66-88)	78 (65-88)	78 (66-89)	0.80

AF: atrial fibrillation; ATA: atrial tachyarrhythmia; BMI: body mass index; creatinine (7.6% N/A); CHA_2_DS_2_-VASc Score (8.1% N/A); CCS-SAF: Canadian Cardiovascular Society Severity of atrial fibrillation; DOAC: direct oral anticoagulant; EHRA: European Heart Rhythm Association; GFR: glomerular filtration rate (7.6% N/A); Q1 and Q3, quartiles (25th and 75th percentiles); SD: standard deviation; TIA: transient ischemic attack; LA: left atrial; LVEF: left ventricular ejection fraction.

### Procedural characteristics

PVI was performed in all patients using RFCA, with a mean fluoroscopy time of 10.6 ± 7.3 minutes and radiation dose of 93 ± 121 mSv (data available for 639 and 622 patients, respectively). The anatomical variation of the left common pulmonary vein (LCPV) was determined in cases where the two left pulmonary veins (PVs) fused at least 10 mm before their common ostium insertion into the left atrium (Figure 1), with 26.6% (n=277) exhibiting this characteristic. Adjunctive posterior wall isolation (PWI - 199 patients [19.1%]) was performed in patients with non-paroxysmal AF patients and in those paroxysmal AF patients in whom high esophageal temperatures prevented PVI.

### Follow-up and atrial tachyarrhythmia recurrence

The mean follow-up time was 2.5 ± 2.3 years (median 1.4 [IQR 1.0 – 3.4] years). Overall, 223 (21.4%) patients had ATA recurrence, 67.3% of which (n=150) occurred in the first year after the procedure. Figure 2 illustrates the rate of ATA recurrence at 12 months after a first-time CA for AF by year of procedure. We observed a significant stepwise decrease in ATA recurrence in the temporal analysis (hazard ratio [HR] 0.94, 95% confidence interval [CI] 0.90 to 0.99; p = 0.01), reaching 1-year recurrence rates as low as 7.5% in 2017. Supplementary Figure 1 depicts the absolute numbers of AF ablations and ATA recurrences according to the year the procedure was performed. Patients with paroxysmal AF had a 12.8% ATA recurrence rate at one year and an overall rate of 18.6%. The ATA recurrence rate for patients with persistent AF was 19.2% at 1 year and 29.8% during long-term follow-up. A survival analysis comparing paroxysmal *versus* non-paroxysmal AF found significantly higher freedom from ATA in paroxysmal AF, as shown in Figure 3 for both (A) 12 months and (B) overall follow-up. Patients with LCPV anatomy had an overall 81.6% freedom from ATA (226/277, p=0.08). Freedom from ATA in paroxysmal and non-paroxysmal AF patients who received adjunctive PWI was 87.2% (82/94, p = 0.11) and 77.1% (81/105, p = 0.04), respectively. Survival analysis comparing ablation techniques with and without CFS catheters shown in Figure 4A found a higher rate of freedom from ATA during follow-up in patients who underwent ablation using CFS catheters (log-rank p = 0.03).

**Figure 2:** – One-year atrial tachyarrhythmia (ATA) recurrence rate following first-time catheter ablation according to the year of procedure; CI: confidence interval; HR: hazard ratio.

**Figure 3:** – Kaplan-Meier curve illustrating atrial tachyarrhythmia (ATA) recurrence in patients with paroxysmal and non-paroxysmal atrial fibrillation (AF) at (A) 12 months and (B) the end of long-term follow-up.

**Figure 4:** – Kaplan-Meier curves for atrial tachyarrhythmia (ATA) recurrence according to (A) contact force-sensing catheter use in first-time catheter ablation and (B) presence of left common pulmonary vein (LCPV) anatomy in patients with persistent atrial fibrillation.

### Univariable and multivariable analysis

Univariable analysis and Cox proportional hazards model to assess predictors of ATA recurrence following CA for AF are presented in Table 2. Independent predictors of ATA recurrence after a first-time ablation included persistent AF at baseline (HR 1.57, 95% CI 1.15 to 2.13; p = 0.004), larger LA diameter (in millimeters) (HR 1.03, 95% CI 1.00 to 1.05; p = 0.033), and patients with an EHRA score class III or IV (HR 1.60, 95% CI 1.18 to 2.18; p = 0.003) as shown in Central Illustration. The procedure year was an independent protective factor, with a 9% relative reduction in recurrence for each new calendar year of the ablation program (Figure 2 and Table 2). These findings were consistent in an analysis restricted to patients with paroxysmal AF, as shown in Supplementary Table 1. A subgroup analysis of ATA recurrence across different age ranges, sex, BMI categories, hypertension, type 2 diabetes, GFR, left ventricular ejection fraction, and LA diameter is shown in Figure 5. Among the subgroups, patients older than 75 years (HR 1.77, 95% CI 1.28-2.45; p = 0.001) and with a larger LA diameter (45-49mm, p = 0.020; >50mm, p = 0.003) had statistically significant worse outcomes regarding ATA. Overall, LA diameter ≥ 45mm (n=211) was associated with more ATA events (HR 1.55, 95% CI 1.15-2.10, p = 0.004).

**Table 2:** – Univariable analysis and Cox proportional hazards model for risk of atrial tachyarrhythmia recurrence after radiofrequency catheter ablation

	Univariable analysis			Multivariable analysis		
	HR	95% CI	p	HR	95% CI	p
Procedure consecutive year	0.94	0.90 – 0.99	0.010	0.91	0.87 – 0.96	<0.001
Persistent atrial fibrillation	1.72	1.30 – 2.28	<0.001	1.57	1.15 – 2.13	0.004
Left atrial diameter enlargement (mm)	1.03	1.01 – 1.05	0.002	1.03	1.00 – 1.05	0.033
EHRA Score of AF Symptoms Class III-IV	1.94	1.26 – 2.97	0.002	1.60	1.18 – 2.18	0.003
Sex	0.82	0.62 – 1.09	0.172			
Age	1.01	1.00 – 1.03	0.027			
Hypertension	1.08	0.82 – 1.42	0.590			
Type 2 diabetes	1.26	0.88 – 1.79	0.205			
Previous stroke	1.74	1.04 – 2.90	0.033			
Beta-blockers use	1.20	0.92 – 1.57	0.172			
Diuretics use	1.38	1.00 – 1.91	0.052			
Left common pulmonary vein	0.91	0.66 – 1.24	0.548			
Use of CFS catheter	0.73	0.55 – 0.97	0.030			
Prior direct cardioversion	1.28	0.96 – 1.70	0.088			

AF: atrial fibrillation; CFS: contact force sensing; EHRA: European Heart Rhythm Association.

**Figure 5:** – Cox proportional hazards model for the risk of atrial tachyarrhythmia (ATA) recurrence after first-time catheter ablation (CA) stratified by subgroups; AF: atrial fibrillation; BMI: body mass index; CA: catheter ablation; CI: confidence interval; HR: hazard ratio; HTN: hypertension; T2DM: type 2 diabetes; GFR: glomerular filtration rat; LVEF: left ventricular ejection fraction; LAD: left atrial diameter.

The multivariable analysis only identified age (HR 1.03, 95% CI 1.00 – 1.05, p = 0.01) as an independent predictor of ATA recurrence when restricted to patients with persistent AF (Supplementary Table 2). Figure 4B illustrates survival analysis in persistent AF patients with and without LCPV (log-rank p = 0.30).

### Safety and adverse events

Over 15 years, among the 1,043 consecutive ablations performed, the complication rate during the index admission was 2.1% (Supplementary Table 3). Notably, patients older than 75 years represented only 0.8% of the overall complications. Severe adverse events included two cases of cardiac tamponade (one successfully managed during the procedure and one requiring cardiac surgery), one non-fatal stroke during admission for index procedure, and one esophageal perforation without fistula successfully treated conservatively. There were no phrenic nerve injuries, clinically relevant pulmonary vein stenosis, or procedure-related deaths (Central Illustration).

## Discussion

This multicenter prospective cohort study evaluated over one thousand consecutive AF patients undergoing first-time CA in Brazil and provides long-term data about efficacy, safety, and predictors of arrhythmia recurrence. The key findings of the current analysis include: (i) overall efficacy and safety were comparable to clinical trials and high-income countries registries in Europe and North America;^14,17^ (ii) long-term efficacy increased over time, with a 9% relative risk reduction of ATA recurrence for each consecutive year that CA was performed; (iii) adopting CFS catheters improved the outcomes after first-time ablation; (iv) most ATA recurrences occurred within one year following ablation; and (v) ATA recurrence was more frequently observed in procedures performed on patients with advanced disease (severe EHRA score of AF symptoms, larger left atrium and persistent AF). Additionally, our findings highlight the low rate of complications of consecutive CAs in a prospective cohort study in Latin America.

International registries play a crucial role in understanding disparities between guidelines and AF management in daily clinical practice. An initial report of the *Atrial Fibrillation Ablation Pilot Registry of the European Society* (EORP-AF) demonstrated 1-year success rates after ablation ranging from 69% to 74.7% in different countries.^24^ The EORP-AF has also contributed significantly to the understanding of real-world data related to AF.^25^ Within the EORP-AF Long-Term Registry, outcomes were documented for 9,663 AF patients based on their antithrombotic therapy.^26^ While 42% and 33% of EORP-AF patients used vitamin K antagonists (VKA) and direct oral anticoagulants (DOACs), respectively, our cohort exhibited a different pattern, with 15% on VKA and 64% on DOACs. Gender-based ablation outcomes have also been reported in this European collaboration, with a similar gender representation to that observed in our study, where only about 30% of patients were female. At baseline, female patients were more symptomatic than male patients, with mean EHRA scores of 2.6 vs. 2.4 in Europe (p<0.001)^27^ and 2.2 vs. 2.0 in Brazil (p<0.001). Importantly, neither study found statistically significant gender-based differences in 12-month recurrence rates (34.4% vs. 34.2% in Europe; 16.1% vs. 13.7% in Brazil, p=0.3), highlighting the need for equitable access to ablation as a treatment option for women.^27^

This study represents the largest cohort study to this date designed to assess ablation outcomes in AF patients in Latin America. Data on Brazilian patients with AF have been recently described in the RECALL Study, although ablation outcomes were not assessed.^21^ The last multicenter registry dedicated to ablation outcomes published in Brazil was conducted by *the Brazilian Society of Cardiac Arrhythmias* between 2005 and 2006.^20^ In this registry, 755 AF patients were included, and a complication rate of 14.3% was reported, which included 1.4% of transient neurologic ischemic events, 0.4% of pulmonary vein stenosis, 3.8% of groin hematomas, and 2.3% other complications. The 2.1% complication rate observed in the current cohort highlights the learning curve associated with ablation procedures and demonstrates how technological advances, especially CFS catheters, have made these procedures safer and more reliable in clinical practice. Similar findings were observed in the largest global cohort study, the *NCDR AFib Ablation Registry*, with a 2.5% complication rate among 76,219 AF patients over five years.^17^

Previous studies have addressed predictors of recurrence after CA for AF. The current analysis demonstrates that persistent AF and larger LA have been consistently reported as independent risk factors.^28,29^ Several scores have been developed to predict rhythm outcomes after AF ablation. The APPLE score (one point for age >65 years, persistent AF, estimated GFR <60 mL/min/1.73 m^2^, LA diameter ≥ 43 mm, LVEF < 50%) had suboptimal performance (AUC = 0.64),^30^ while the AFA-Recur web calculator based on a random forest model of 19 variables achieved an acceptable discriminative performance (AUC 0.72).^31^ Our Cox model also incorporated the EHRA score of AF symptoms class III-IV as an independent predictor of risk. The EHRA AF score is commonly used to assess clinical response following CA,^32^ and might also signal the severity and longer duration of the disease. Compared to previous registries, our cohort also showed higher ATA rates following CA in older patients, although there was no significant difference in outcomes across BMI categories.^33,34^

It is reasonable to propose that paroxysmal AF patients exposed to longer arrhythmia burdens experience progressive remodeling of the atrium. This possibly leads to worsening of underlying atriopathy and progression to the persistent or more severe forms of the disease. Ultimately, this expected progression of AF leads to worse clinical outcomes in procedures performed later in the natural history of the arrhythmia. This proposition was substantiated in the EARLY-AF trial, where paroxysmal AF patients were followed for three years.^35^ This study revealed that patients who underwent initial CA had a lower progression to persistent AF and fewer ATA recurrences when compared with those treated solely with antiarrhythmic drugs. While it seems evident that earlier ablations could yield better results, achieving high efficacy in persistent AF ablations remains a challenge. Adjunctive ablation sites, such as PWI, have been suggested recently as a potential strategy for managing this challenging condition.^36,37^ In the current cohort, PWI was performed in approximately one-fifth of enrolled patients at the operator's discretion but was not an independent predictor of ATA recurrence.

### Strengths and limitations

Our cohort comprises consecutive AF patients undergoing their first-time ablation, making it Latin America’s largest dataset dedicated to evaluating the safety and efficacy of CA for AF. These findings are particularly relevant in the context of LMICs, providing valuable insights into real-world clinical safety. Residual confounding is a potential concern, as we lacked data to adjust for AF duration. We did not analyze the outcomes of redo ablations in this study. This multicenter cohort was conducted only in private centers and might not reflect the reality of public centers in Brazil. Most patients were white and did not represent the population in Latin America. Additionally, patients were censored at the last follow-up, which could have underestimated the ATA recurrence rate.

## Conclusion

In the largest cohort study in Latin America of consecutive first-time ablations for AF, ATA recurrence was associated with interventions conducted at later stages of the disease, highlighting the significance of early intervention for improved clinical outcomes. Peri-procedural complications and ATA recurrence rates were comparable to those in high-income countries, underscoring the global applicability of CA for AF management. Overall, these data highlight the outstanding performance of CA in AF management in Latin American centers, suggesting that this treatment option should be expanded to the public health system in Brazil.
